# Annealing effect on Sb_2_S_3_-TiO_2_ nanostructures for solar cell applications

**DOI:** 10.1186/1556-276X-8-89

**Published:** 2013-02-19

**Authors:** Yitan Li, Lin Wei, Ruizi Zhang, Yanxue Chen, Liangmo Mei, Jun Jiao

**Affiliations:** 1 School of Physics and State Key Laboratory of Crystal Materials, Shandong University, Jinan, 250100, People's Republic of China; 2 School of Information Science and Engineering, Shandong University, Jinan, 250100, People's Republic of China; 3 Department of Mechanical and Materials Engineering, Portland State University, P.O. Box 751, Portland, OR, 97207-0751, USA; 4 Department of Physics, Portland State University, P.O. Box 751, Portland, OR, 97207-0751, USA

**Keywords:** TiO_2_, Sb_2_S_3_, Nanorod, Solar cells, Annealing effect

## Abstract

Nanostructures composited of vertical rutile TiO_2_ nanorod arrays and Sb_2_S_3_ nanoparticles were prepared on an F:SnO_2_ conductive glass by hydrothermal method and successive ionic layer adsorption and reaction method at low temperature. Sb_2_S_3_-sensitized TiO_2_ nanorod solar cells were assembled using the Sb_2_S_3_-TiO_2_ nanostructure as the photoanode and a polysulfide solution as an electrolyte. Annealing effects on the optical and photovoltaic properties of Sb_2_S_3_-TiO_2_ nanostructure were studied systematically. As the annealing temperatures increased, a regular red shift of the bandgap of Sb_2_S_3_ nanoparticles was observed, where the bandgap decreased from 2.25 to 1.73 eV. At the same time, the photovoltaic conversion efficiency for the nanostructured solar cells increased from 0.46% up to 1.47% as a consequence of the annealing effect. This improvement can be explained by considering the changes in the morphology, the crystalline quality, and the optical properties caused by the annealing treatment.

## Background

Dye-sensitized solar cells (DSSCs) pioneered by O'Regan and Grätzel have been intensively investigated as a promising photovoltaic cell all over the world [1-5]. Until now, photovoltaic conversion efficiency of up to 11% has been reported for DSSCs in the laboratory [6]. Although the conversion efficiency is impressive, the expense of the dye required to sensitize the solar cell is still not feasible for practical applications. Therefore, it is critical to tailor the materials to be not only cost effective but also long lasting. Recently, the utilization of narrow-bandgap semiconductors as a light-absorbing material, in place of conventional dye molecules, has drawn much attention. Inorganic semiconductors have several advantages over conventional dyes: (1) The bandgap of semiconductor nanoparticles can be easily tuned by size over a wide range to match the solar spectrum. (2) Their large intrinsic dipole moments can lead to rapid charge separation and large extinction coefficient, which is known to reduce the dark current and increase the overall efficiency. (3) In addition, semiconductor sensitizers provide new chances to utilize hot electrons to generate multiple charge carriers with a single photon. These properties make such inorganic narrow-bandgap semiconductors extremely attractive as materials for photovoltaic applications.

Recently, a range of nano-sized semiconductors has been investigated in photovoltaic applications including CdS [7-9], CdSe [10-13], Ag_2_S [14], In_2_S_3_[15], PbS [16], Sb_2_S_3_[17], Cu_2_O [18], as well as III-VI quantum ring [19]. Among these narrow-bandgap semiconductors, Sb_2_S_3_ has shown much promise as an impressive sensitizer due to its reasonable bandgap of about 1.7 eV, exhibiting a strong absorption of the solar spectrum. The use of Sb_2_S_3_ nanoparticles, which may produce more than one electron–hole pair per single absorbed photon (also known as multiple exciton generation), is a promising solution to enhance power conversion efficiency. Furthermore, the creation of a type-II heterojunction by growing Sb_2_S_3_ nanoparticles on the TiO_2_ surface greatly enhances charge separation. All of these effects are known to increase the exciton concentration, lifetime of hot electrons, and therefore, the performance of sensitized solar cells. Limited research has previously been carried out with Sb_2_S_3_-TiO_2_ nanostructure for solar cell applications [20-22]. A remarkable performance was obtained in both liquid cell configuration and solid configuration. These findings were based on the use of porous nanocrystalline TiO_2_ particles; however, very little research has been conducted using single-crystalline TiO_2_ nanorod arrays. Compared with conventional porous polycrystalline TiO_2_ films, single-crystalline TiO_2_ nanorods grown directly on transparent conductive oxide electrodes provide an ideal alternative solution by avoiding particle-to-particle hopping that occurs in polycrystalline films, thereby increasing the photocurrent efficiency. Further enhancements in solid Sb_2_S_3_-sensitized solar cells demand a deeper understanding of the main parameters determining photoelectric behavior while also requiring additional research and insight into the electrical transporting process in these nanostructures.

**Figure 1:**
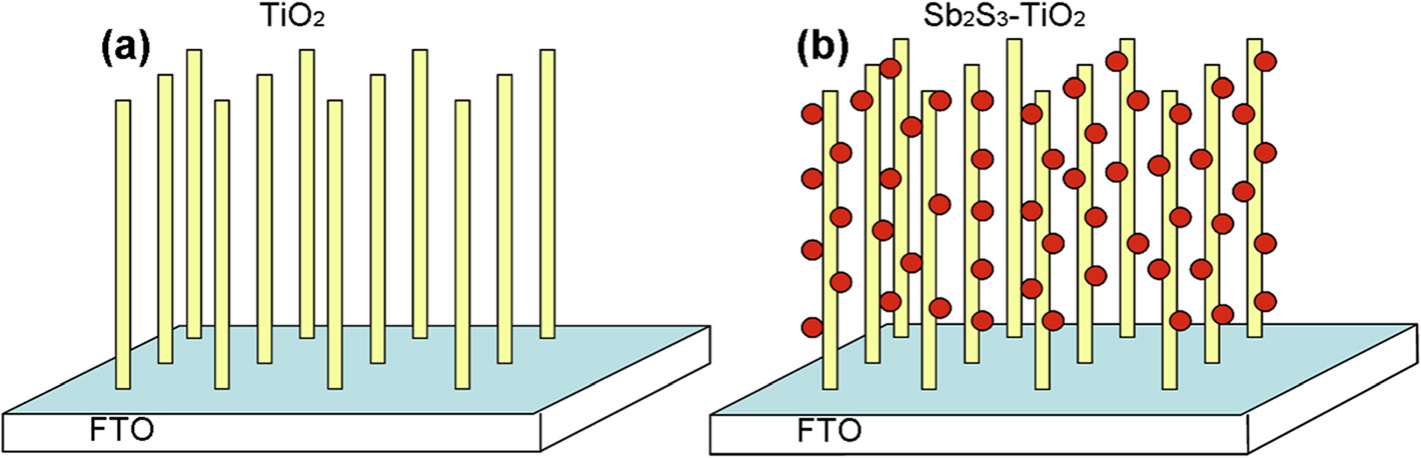
Schematic of (a) bare TiO_2_ nanorod arrays on FTO and (b) Sb_2_S_3_-TiO_2_ nanostructure on FTO.

In our present research study, Sb_2_S_3_ semiconductor nanoparticles and single-crystalline rutile TiO_2_ nanorod arrays were combined to perform as a photoanode for a practical nanostructured solar cell (as depicted in Figure 1). The annealing effect on the photovoltaic performance and optical property of Sb_2_S_3_-TiO_2_ nanostructures was studied systematically, and the optimal temperature of 300°C was confirmed. After annealing, apparent changes of morphological, optical, and photovoltaic properties were observed. The photovoltaic conversion efficiency of solar cell assembled using annealed Sb_2_S_3_-TiO_2_ nanostructure demonstrated a significant increase of 219%, compared with that based on as-made Sb_2_S_3_-TiO_2_ nanostructure.

## Methods

### Growth of single-crystalline rutile TiO_2_ nanorod arrays by hydrothermal process

TiO_2_ nanorod arrays were grown directly on fluorine-doped tin oxide (FTO)-coated glass using the following hydrothermal methods: 50 mL of deionized water was mixed with 40 mL of concentrated hydrochloric acid. After stirring at ambient temperature for 5 min, 400 μL of titanium tetrachloride was added to the mixture. The feedstock, prepared as previously described, was injected into a stainless steel autoclave with a Teflon lining. The FTO substrates were ultrasonically cleaned for 10 min in a mixed solution of deionized water, acetone, and 2-propanol with volume ratios of 1:1:1 and were placed at an angle against the Teflon liner wall with the conducting side facing down. The hydrothermal synthesis was performed by placing the autoclave in an oven and keeping it at 180°C for 2 h. After synthesis, the autoclave was cooled to room temperature under flowing water, and the FTO substrates were taken out, washed extensively with deionized water, and dried in open air.

### Deposition of Sb_2_S_3_ nanoparticles with successive ionic layer adsorption and reaction method and annealing treatment

Successive ionic layer adsorption and reaction (SILAR) method was used to prepare Sb_2_S_3_ semiconductor nanoparticles. In a typical SILAR cycle, the F:SnO_2_ conductive glass, pre-grown with TiO_2_ nanorod arrays, was dipped into the 0.1 M antimonic chloride ethanol solution for 5 min at 50°C. Next, the F:SnO_2_ conductive glass was rinsed with ethanol and then dipped in 0.2 M sodium thiosulfate solution for 5 min at 80°C and finally rinsed in water. This entire SILAR process was repeated for 10 cycles. After the SILAR process, samples were annealed in N_2_ flow at varied temperatures from 100°C to 400°C for 30 min. After annealing, a color change was noted in the Sb_2_S_3_-TiO_2_ nanostructured samples, which were orange before annealing and gradually turned blackish as the annealing temperature increased.

### Characterization of the Sb_2_S_3_-TiO_2_ nanostructures

The crystal structure of the Sb_2_S_3_-TiO_2_ samples were examined by X-ray diffraction (XD-3, PG Instruments Ltd., Beijing, China) with CuKα radiation (*λ* = 0.154 nm) at a scan rate of 2°/min. X-ray tube voltage and current were set at 40 kV and 30 mA, respectively. The surface morphology of the Sb_2_S_3_-TiO_2_ nanostructures was examined by scanning electron microscopy (SEM; FEI Sirion, FEI Company, Hillsboro, OR, USA). The optical absorption spectra were obtained using a dual beam UV-visible spectrometer (TU-1900, PG Instruments, Ltd.).

### Solar cell assembly and performance measurement

Solar cells were assembled using a Sb_2_S_3_-TiO_2_ nanostructure as the photoanode. Pt counter electrodes were prepared by depositing an approximately 20-nm Pt film on FTO glass using magnetron sputtering. A 60-μm-thick sealing material (SX-1170-60, Solaronix SA, Aubonne, Switzerland) with a 3 × 3 mm aperture was pasted onto the Pt counter electrodes. The Pt counter electrode and the Sb_2_S_3_-TiO_2_ sample were sandwiched and sealed with the conductive sides facing inward. A polysulfide electrolyte was injected into the space between the two electrodes. The polysulfide electrolyte was composed of 0.1 M sulfur, 1 M Na_2_S, and 0.1 M NaOH which were dissolved in distilled water and stirred at 80°C for 2 h.

A solar simulator (Model 94022A, Newport, OH, USA) with an AM1.5 filter was used to illuminate the working solar cell at light intensity of one sun illumination (100 mW/cm^2^). A source meter (2400, Keithley Instruments Inc., Cleveland, OH, USA) was used for electrical characterization during the measurements. The measurements were carried out using a calibrated OSI standard silicon solar photodiode.

## Results and discussion

### Morphology and crystal structure of Sb_2_S_3_-TiO_2_ nanostructure

**Figure 2:**
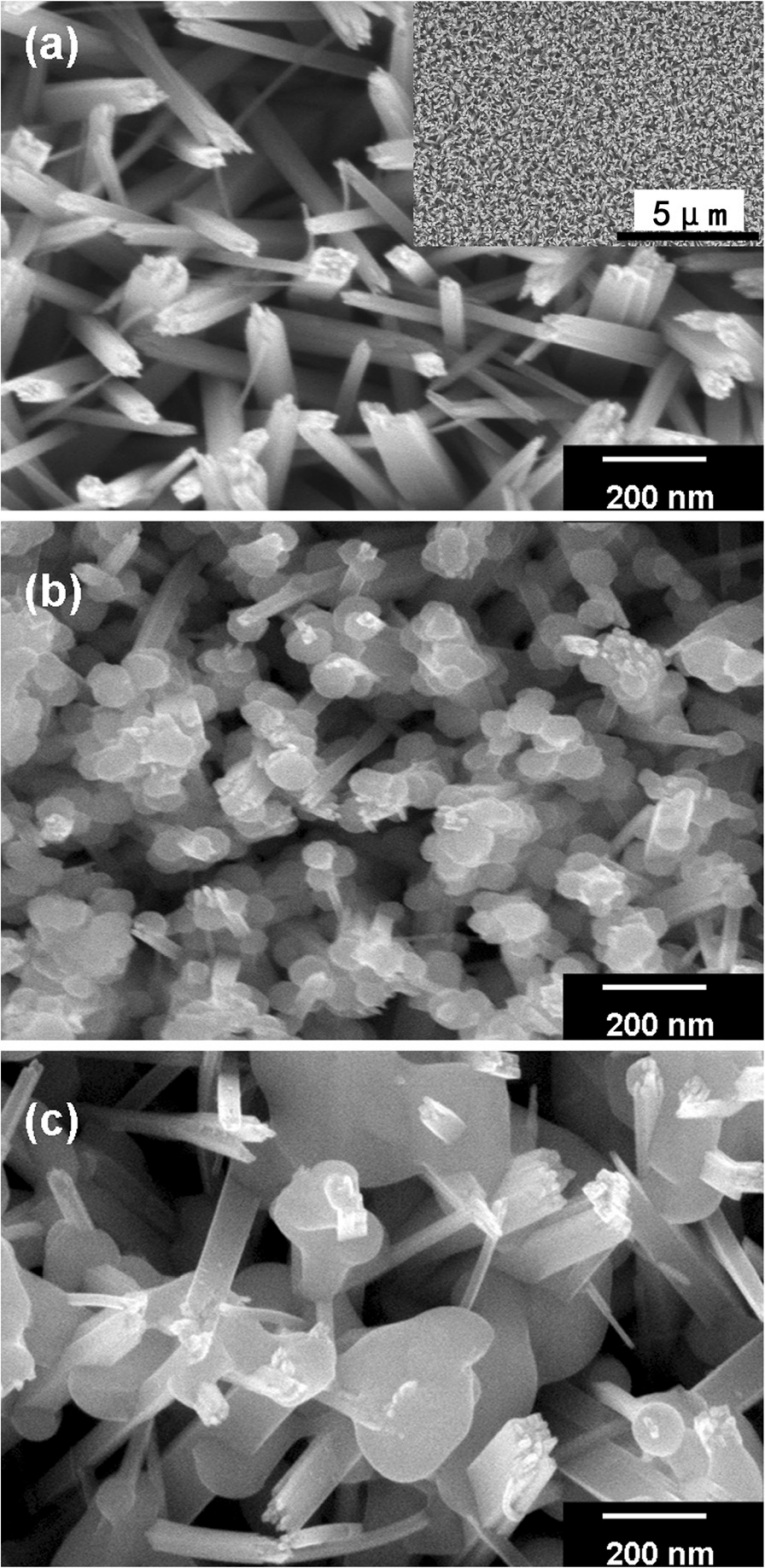
**Typical top-view SEM images of TiO_2_ nanorod arrays and Sb_2_S_3_-TiO_2_ nanostructures. **(**a**) SEM image of a TiO_2_ nanorod array grown on SnO_2_:F substrate by hydrothermal process. Inset: A low-magnification SEM image of the same sample. (**b**) SEM image of the as-grown Sb_2_S_3_-TiO_2_ nanostructures. (**c**) SEM image of Sb_2_S_3_-TiO_2_ nanostructures annealed at 300°C for 30 min.

The morphology of the rutile TiO_2_ nanorod arrays is shown in Figure 2a. The SEM images clearly show that the entire surface of the FTO glass substrate was uniformly covered with ordered TiO_2_ nanorods, and the nanorods were tetragonal in shape with square top facets. This nanorod array presented an easily accessed open structure for Sb_2_S_3_ deposition and a higher hole transferring speed for the whole solar cell. No significant changes in nanorod array morphology were observed after annealing at 400°C. As-synthesized Sb_2_S_3_-TiO_2_ nanostructure is shown in Figure2b, indicating a combination of the Sb_2_S_3_ nanoparticles and TiO_2_ nanorods. The Sb_2_S_3_-TiO_2_ nanostructure after annealing at 300°C for 30 min is shown in Figure 2c. Compared to the CdS-TiO_2_ nanostructure, in which 5-to 10-nm CdS nanoparticles distributed uniformly on the TiO_2_ nanorod [9], the as-deposited Sb_2_S_3_ particles differed with a larger diameter of approximately 50 nm and often covered several TiO_2_ nanorods. This structural phenomenon was observed much more so in the annealed sample, where at least some melting of the low melting point (550°C) Sb_2_S_3_ clearly occurred. After the annealing treatment, the size of Sb_2_S_3_ particles increased, which enabled the Sb_2_S_3_ particles to closely contact the TiO_2_ nanorod surface. This solid connection between Sb_2_S_3_ nanoparticles and the TiO_2_ nanorods was beneficial to the charge separation and improved the overall properties of the sensitized solar cells.

**Figure 3:**
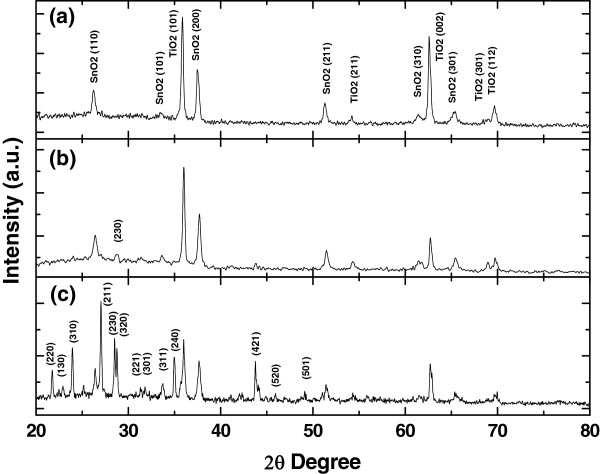
**XRD patterns.** The bare TiO_2_ nanorod arrays (**a**), the as-grown Sb_2_S_3_-TiO_2_ nanostructure electrode (**b**), and the annealed Sb_2_S_3_-TiO_2_ nanostructure electrode under 300°C (**c**).

X-ray diffraction (XRD) patterns of the bare TiO_2_ nanorod array, the as-synthesized Sb_2_S_3_-TiO_2_ nanostructure, and the annealed nanostructure are shown in Figure 3. Note in Figure 3a that the TiO_2_ nanorod arrays grown on the FTO-coated glass substrates had a tetragonal rutile structure (JCPDS no. 02–0494), which may be attributed to the small lattice mismatch between FTO and rutile. The as-synthesized Sb_2_S_3_-TiO_2_ nanostructure exhibited a weak diffraction peak (Figure 3b) at 2*θ* = 28.7°, corresponding to the (230) plane of orthorhombic Sb_2_S_3_. As the annealing temperature increased, more diffraction peaks were observed, and the peaks became more distinct at the same time. Figure 3c shows the XRD pattern of the nanostructure annealed at less than 300°C. All of the reflections were indexed to an orthorhombic phase of Sb_2_S_3_ (JCPDS no. c-74-1046) [23]. The shape of the diffraction peaks indicates that the product was well crystallized.

### Optical property of the Sb_2_S_3_-TiO_2_ nanostructures

**Figure 4:**
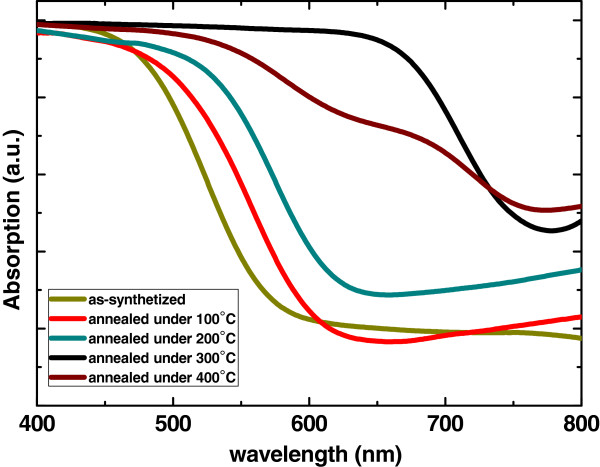
**Optical absorption spectra of Sb_2_S_3_-TiO_2_ nanostructure samples.** Before (green spectrum) and after being annealed at 100°C (red spectrum), 200°C (blue-green spectrum), 300°C (black spectrum), and 400°C (brown spectrum).

The UV-visible absorption spectra of Sb_2_S_3_-TiO_2_ nanostructure samples are shown in Figure 4. An optical bandgap of 2.25 eV is estimated for the as-synthesized Sb_2_S_3_ nanoparticles from the absorption spectra, which exhibits obvious blueshift compared with the value of bulk Sb_2_S_3_. After being annealed at 100°C, 200°C, and 300°C for 30 min, the bandgap of Sb_2_S_3_ nanoparticles was red shifted to 2.19 eV (565 nm), 2.13 eV (583 nm), and 1.73 eV (716 nm), respectively. When annealed at 400°C, the absorption spectra deteriorated, which may be attributed to the oxidation as well as the evaporation of the Sb_2_S_3_ nanoparticles. The Sb_2_S_3_-TiO_2_ nanostructure annealed at 300°C shows an enhanced absorption in the visible range, which is of great importance for solar cell applications and will result in higher power conversion efficiency. As shown by the XRD patterns and SEM images, this red shift in the annealed samples may be explained by the annealing-induced increase in particle size at the elevated temperatures. The annealing effect on the optical absorption spectra of bare TiO_2_ nanorod arrays was also studied (not included here). No obvious difference was found between the samples with and without annealing treatment. This result suggests that although annealing changes the morphology and crystallinity of Sb_2_S_3_ nanoparticles, it does not significantly affect the optical property of TiO_2_ nanorod arrays.

### Photovoltaic performance of the solar cell based on Sb_2_S_3_-TiO_2_ nanostructure

**Figure 5:**
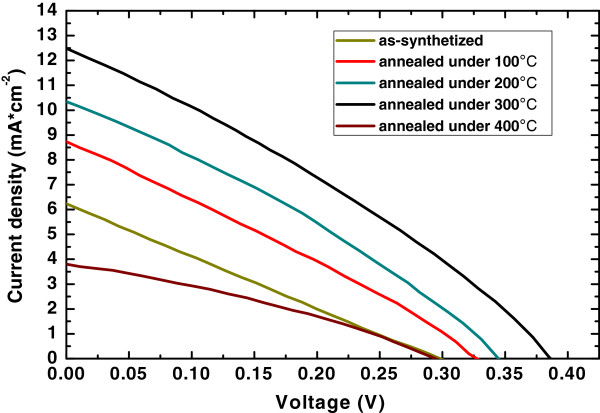
***I*-*V *curves for the solar cells assembled using Sb_2_S_3_-TiO_2_ nanostructures annealed under varied temperature.**

The photocurrent-voltage (*I*-*V*) performances of the solar cells assembled using Sb_2_S_3_-TiO_2_ nanostructures annealed under different temperatures are shown in Figure 5. The *I*-*V* curves of the samples were measured under one sun illumination (AM1.5, 100 mW/cm^2^). Compared with the solar cell based on as-grown Sb_2_S_3_-TiO_2_ nanostructure, the solar cell performances correspondingly improved as the annealing temperatures increased from 100°C to 300°C. The open-circuit voltage (*V*_oc_) improved from 0.3 up to 0.39 V, and the short-circuit current density (*J*_sc_) improved from 6.2 up to 12.1 mA/cm^2^. A power conversion efficiency of 1.47% for the sample with annealing treatment was obtained, indicating an increase of 219% (as compared to the 0.46% for the as-grown sample) as a consequence of the annealing treatment. The photovoltaic performance of annealed Sb_2_S_3_-TiO_2_ nanostructured solar cell under 400°C deteriorated, which coincides with the absorption spectrum. Detailed parameters of the solar cells extracted from the *I*-*V* characteristics are listed in Table 1.

**Table 1:** Parameters of Sb_2_S_3_-TiO_2_ nanostructured solar cells annealed at different temperatures

	***V***_**oc**_**(V)**	***J***_**sc**_**(mA/cm**^**2**^**)**	**FF (%)**	***η *(%)**
As-synthesized Sb_2_S_3_-TiO_2_	0.30	6.10	0.25	0.46
Sb_2_S_3_-TiO_2_ under 100°C	0.33	8.65	0.28	0.79
Sb_2_S_3_-TiO_2_ under 200°C	0.34	10.32	0.31	1.10
Sb_2_S_3_-TiO_2_ under 300°C	0.39	12.15	0.31	1.47
Sb_2_S_3_-TiO_2_ under 400°C	0.29	3.82	0.32	0.36

*V*_oc_, open-circuit voltage; *J*_sc_, integral photocurrent density; FF, fill factor; *η*, power conversion efficiency.

This significant improvement of the photovoltaic performance obtained for annealed Sb_2_S_3_-TiO_2_ nanostructured solar cells is explained by the following reasons: (1) An enhanced absorption of sunlight caused by the red shift of the bandgap will result in an enhanced current density. (2) Increase of Sb_2_S_3_ grain size by annealing will reduce the particle-to-particle hopping of the photo-induced carrier. This hopping may occur in an as-grown nanostructure with Sb_2_S_3_ nanoparticles. (3) Improvement of crystal quality of the Sb_2_S_3_ nanoparticles by annealing treatment will decrease the internal defects, which can reduce the recombination of photoexcited carriers and result in higher power conversion efficiency. (4) Good contact between the Sb_2_S_3_ nanoparticles and the TiO_2_ nanorod is formed as a result of high-temperature annealing. Such a superior interface between TiO_2_ and nanoparticles can inhibit the interfacial recombination of the injected electrons from TiO_2_ to the electrolyte, which is also responsible for its higher efficiency.

Our findings suggest the possible use of 3D nanostructure material grown by a facile hydrothermal method for sensitized solar cell studies. The drawback of this type of solar cell is a rather poor fill factor, which limits the energy conversion efficiency. This low fill factor may be ascribed to the lower hole recovery rate of the polysulfide electrolyte, which leads to a higher probability for charge recombination [24]. To further improve the efficiency of these nanorod array solar cells, we advise that a new hole transport medium with suitable redox potential and low electron recombination at the semiconductor and electrolyte interface should be developed. Moreover, as reported by Soel et al., other contributions such as the counter electrode material may also influence the fill factor [25].

## Conclusions

With a facile hydrothermal method, the single-crystalline TiO_2_ nanorod arrays were successfully grown on fluorine-doped tin oxide glass. Next, Sb_2_S_3_ nanoparticles were deposited by successive ionic layer adsorption and reaction method to form a Sb_2_S_3_-TiO_2_ nanostructure for solar cell applications. Annealing treatment was conducted under varied temperatures, and the optimal annealing temperature of 300°C was obtained. Obvious enhancement in visible light absorption was observed for the annealed samples. The photovoltaic performance for solar cells based on annealed Sb_2_S_3_-TiO_2_ nanostructure shows an increase of up to 219% in power conversion efficiency.

## Competing interests

The authors declare that they have no competing interests.

## Authors' contributions

YL carried out the preparation of Sb_2_S_3_-TiO_2_ nanostructured solar cells and drafted the manuscript. LW conducted the optical absorption spectra and the *I*-*V* measurements. RZ carried out the preparation of TiO_2_ nanorod arrays and the XRD measurements. YC carried out the SEM characterization and supervised the work. LM and JJ analyzed the results and finalized the manuscript. All authors read and approved the final manuscript.
